# Complex dental wear analysis reveals dietary shift in Triassic placodonts (Sauropsida, Sauropterygia)

**DOI:** 10.1186/s13358-024-00304-x

**Published:** 2024-02-05

**Authors:** Kinga Gere, András Lajos Nagy, Torsten M. Scheyer, Ingmar Werneburg, Attila Ősi

**Affiliations:** 1 Institute of Geography and Earth Sciences, Department of Paleontology, ELTE Eötvös Loránd University, Pázmány Péter Sétány 1/C, 1117 Budapest, Hungary; 2 Department of Propulsion Technology, Széchenyi István University, Egyetem Tér 1, 9026 Győr, Hungary; 3 Universität Zürich, Paläontologisches Institut, Karl Schmid-Strasse 4, CH-8006 Zurich, Switzerland; 4 Senckenberg Centre for Human Evolution and Palaeoenvironment, Universität Tübingen, Sigwartstraße 10, 72076 Tübingen, Germany; 5 Fachbereich Geowissenschaften an der Eberhard Karls Universität Tübingen, 72074 Tübingen, Germany; 6 Hungarian Natural History Museum, Ludovika Tér 2, 1083 Budapest, Hungary

**Keywords:** Placodontia, Tooth wear analysis, Durophagy, Marine reptile

## Abstract

Placodonts were durophagous reptiles of the Triassic seas with robust skulls, jaws, and enlarged, flat, pebble-like teeth. During their evolution, they underwent gradual craniodental changes from the Early Anisian to the Rhaetian, such as a reduction in the number of teeth, an increase in the size of the posterior palatal teeth, an elongation of the premaxilla/rostrum, and a widening of the temporal region. These changes are presumably related to changes in dietary habits, which, we hypothesise, are due to changes in the type and quality of food they consumed. In the present study, the dental wear pattern of a total of nine European Middle to Late Triassic placodont species were investigated using 2D and 3D microwear analyses to demonstrate whether there could have been a dietary shift or grouping among the different species and, whether the possible changes could be correlated with environmental changes affecting their habitats. The 3D analysis shows overlap between species with high variance between values and there is no distinct separation. The 2D analysis has distinguished two main groups. The first is characterised by low number of wear features and high percentage of large pits. The other group have a high feature number, but low percentage of small pits. The 2D analysis showed a correlation between the wear data and the size of the enlarged posterior crushing teeth. Teeth with larger sizes showed less wear feature (with higher pit ratio) but larger individual features. In contrast, the dental wear facet of smaller crushing teeth shows more but smaller wear features (with higher scratch number). This observation may be related to the size of the food consumed, i.e., the wider the crown, the larger food it could crush, producing larger features. Comparison with marine mammals suggests that the dietary preference of *Placochelys*, *Psephoderma* and *Paraplacodus* was not exclusively hard, thick-shelled food. They may have had a more mixed diet, similar to that of modern sea otters. The diet of *Henodus* may have included plant food, similar to the modern herbivore marine mammals and lizards.

**Supplementary Information:**

The online version contains supplementary material available at 10.1186/s13358-024-00304-x.

## Introduction

Placodonts are highly morphologically specialised extinct marine reptiles of the Triassic seas. Their geographical distribution covers the epicontinental sea of the Germanic Basin (e.g.Drevermann, 1933; Hagdorn & Rieppel, 1998; Nosotti & Pinna, 1996; Rieppel, 2001; von Huene, 1946; Westphal, 1967, 1988), the carbonate platform of the Western Tethys (e.g.Buffetaut & Novak, 2008; Gere et al., 2020; Kuhn-Schnyder, 1974; Peyer, 1931a, 1931b; Pinna, 1992) and the Eastern Tethys (Jiang et al., 2008; Li, 2000; Li & Rieppel, 2002; Wang et al., 2019; Zhao et al., 2008). In their evolutionary history, numerous morphological changes can be detected, the most characteristic of which are related to their feeding ecology, such as the size, the shape and the number of the teeth, and the skull morphology with the shape of the rostrum and the temporal region in particular.

Most of the placodont species have highly specialised crushing dentition with globular or flattened button-like teeth. The maxilla, the premaxilla, the palatine and the dentary of a placodont can bear teeth, among which the last two bones have extremely enlarged teeth. In early branching taxa, teeth were angular and dentition is characterised by a relatively high tooth number (a total of 10–14 pairs of teeth in premaxilla, maxilla and palate) as seen in the Middle Triassic *Placodus gigas* and *Paraplacodus broilii*. In Late Triassic taxa, the teeth became rounded and reduced in number. Especially, the Late Triassic placodonts lost the premaxillary teeth (*Placochelys placodonta*, *Psephoderma alpinum*, *Macroplacus raeticus*) or the dentition is extremely reduced to only one pair of palatine/dentary teeth (*Henodus chelyops*) (Crofts et al., 2017; Mazin, 1989; Pommery et al., 2021; Reif & Stein, 1999; Rieppel, 1995, 2000, 2001). In addition, the tooth crown morphology became more complex from early branching placodonts to the more highly nested cyamodontoids, especially placochelyids (Crofts et al., 2017). Neenan et al., 2014 studied the tooth replacement pattern of placodont morphotypes mainly on crania and lower jaws, where it was possible, and they pointed out that the pattern follows the phylogenetic trend. *Placodus gigas* has many replacement teeth and little or no pattern, while cyamodontoids have fewer replacement teeth and unilaterally, continuously maintaining a functional crushing area. Against these, the placochelyids (studied *Psephoderma alpinum* and the Chinese *Psephochelys polyosteoderma*) show only one or two replacement teeth (see also *Henodus chelyops*: Pommery et al., 2021). Rieppel (2002) also supported the durophagous lifestyle of placodonts based on the reconstruction of jaw adductor musculature and noted differences between each morphotype.

The basic tooth morphology with enlarged, flattened teeth indicate a durophagous diet (e.g., gastropods, bivalves, brachiopods, crustaceans). However, there is a few evidence including stomach contents preserved with placodont skeletons or biting marks on contemporaneous invertebrate fossils to confirm these hypothetic habits (Yiang et al., 2008). Examples of extant animals show that dental morphology alone might not always give an unambiguous signal, as exemplified by the lizard species *Dicrodon guttulatum* and *Teius teyou* (Brizuela & Kosma, 2017). These taxa have similar tooth morphology, but the former is herbivorous (Vitt, 2004), whereas the latter is insectivorous or omnivorous (Presch, 1974, Meltsrom, 2017). The cranial and dental morphologies of placodonts are significantly varied from relatively basal member *Psephochelys polyosteoderma* to one of the most derived taxa *Henodus chelyops*. These suggest various specializations in their feeding mode (Neenan et al., 2013; Rieppel, 2002) and food type during the evolutionary history of the group. So far, the dentition and feeding mechanism of *Henodus chelyops* has been studied in more detail (Pommery et al., 2021), and Crofts et al. (2017) investigated the radius of curvature of the occlusal surface on tooth in placodonts.

Dental wear analysis has not yet been examined on the placodonts. Dental wear studies (2D and 3D) are a suitable method to detect major differences in the type of the food consumed (e.g.DeSantis, 2016; DeSantis et al., 2012; Pedraza et al., 2022; Solounias & Hayek, 1993) and to interpret their possible spatial and temporal changes in an evolutionary context. Although these examinations are most commonly used for mammals, they also work well for reptiles (Ősi & Weishampel, 2009; Mallon & Anderson, 2014; Ősi et al., 2017; Winkler et al., 2019) and fishes (Purnell & Darras, 2016; Purnell et al., 2012). In extant lizards, it has been possible to distinguish between the molluscivore, the herbivore or the carnivore (Winkler et al., 2019). Similarly, in fish there are significant differences between specialist and generalist durophages (Purnell & Darras, 2016). Using the results of these studies in marine mammals, the aim of this study is to use this method to demonstrate possible differences in the diet of placodonts.

## Materials and methods

Institutional abbreviations: BSP Bayerische Staatssammlung für Paläontologie und Historische Geologie München, Germany; GPIT Geologisch–Paläontologisches Institut, Universität Tübingen, Germany; SZTFH the Department of Collections of the Supervisory Authority for Regulatory Affairs, formerly the Collection of the Hungarian Geological Institute, Budapest, Hungary; MTM Hungarian Natural History Museum, Budapest, Hungary**;** PIMUZ Paläontologisches Institut, Universität Zürich, Switzerland**;** SMF Senckenberg Forschungsinstitut und Naturmuseum Frankfurt, Germany**;** SMNS Staatliches Museum für Naturkunde Stuttgart, Germany.

### Material and sample preparation

**Fig. 1:**
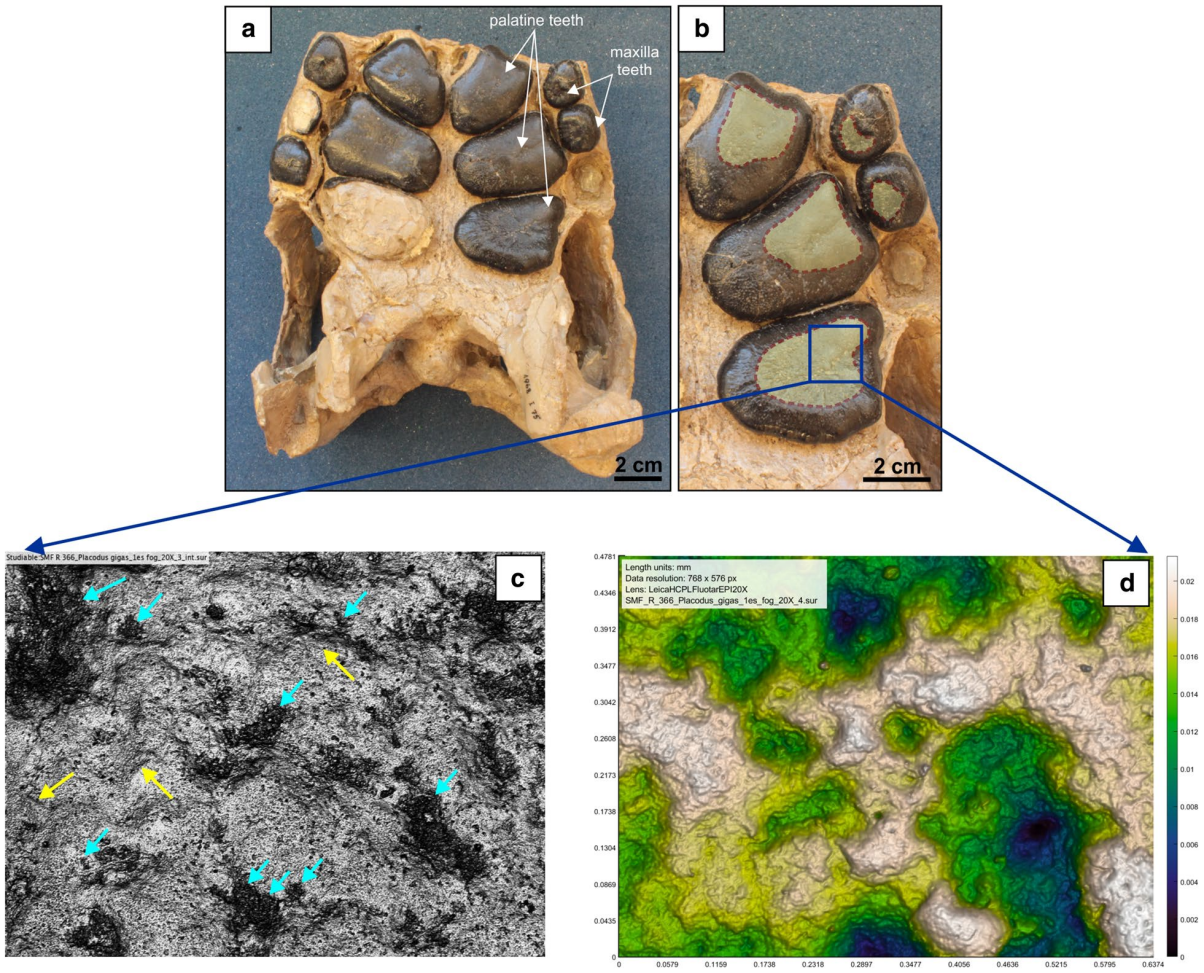
Methodology **a** BSP 1968 I 75 partial cranium of *Placodus gigas* showing the enlarged palatine and maxillary teeth in palatal view; **b** left palatine and maxillary teeth in occlusal view. The dashed lines show the worn surfaces. The photosimulations of the microwear analysis were taken from the area marked with blue square (distal palatine teeth); **c** two-dimensional photosimulation of the worn surface in occlusal view. The blue arrows show small and large pits, while the yellow arrows indicate scratches. **d** Three-dimensional worn dental surface. The area is 637 × 468 μm (= 20 × magnification) in both cases

The present study focuses on the European placodont material, mainly the following taxa: *Placodus gigas*, *Paraplacodus broilii*, *Cyamodus* cf. *rostratus*, *Cyamodus hildegardis*, *Cyamodus kuhnschnyderi*, *Cyamodus* sp., *Placochelys placodonta*, *Henodus chelyops*, *Psephoderma alpinum* and *Macroplacus raeticus*. Supplementary 1 shows the specimens (50 individuals) used in this study. All of the teeth of the specimens are from adults and the enamel was not broken or damaged during fossilisation. The dentition of placodonts is not uniform. There are significant differences in the type and number of teeth. *Psephoderma alpinum*, *Placochelys placodonta* and *Macroplacus raeticus* do not have premaxillary teeth. The latter has fragmented cranium with incomplete premaxilla. However, there is also a difference in the number of maxillary and palatal/dentary teeth. The earlier species, such as *Paraplacodus broilii* and *Placodus gigas* have more teeth than later forms (e.g., *Placochelys*). The most distinctive is *Henodus chelyops*, which has a pair of posterior palatal/dentary teeth. Furthermore, the function of the anterior and posterior teeth is different. While the anterior teeth are for grasping, the posterior teeth are for chewing and processing food. Usually posterior teeth are the most worn, where tooth–food and tooth–tooth contact was most proficient. For this reason, and to ensure uniformity of the sampling, the enlarged palatal/dentary teeth were selected for examination (Fig. 1a, b). In situ and isolated teeth were also examined, the latter have not been determined to be dentary or palatal, but definitely posterior, enlarged teeth. Teeth of some extant marine mammals were used to compare with those of placodonts, including three specimens of sea otters (*Enhydra lutris*), one manatee (*Trichechus manatus*) and one dugong (*Dugong dugon)*, housed in the Mammal Collection of the Hungarian Natural History Museum (Additional file 1).

A widely used method for microwear analysis was applied to make casts from the tooth crowns (e.g.Merceron & Madelaine, 2006; Merceron et al., 2005; Solounias & Semprebon, 2002). First, the tooth surfaces were cleaned with alcoholic cotton swabs (ethanol) from dirt. Any remaining cotton fibres were removed under a microscope using a blunt toothpick without damaging the enamel. After preparatory work, the moulds were made using a high resolution (< 0.1 μm) polyvinylsiloxane dental impression material (Heraeus Kulzer Provil Novo light regular set). Then, the silicone moulds were poured using EpoTek301 epoxy resin.

The specific surfaces on the moulds were imaged on a Leica DCM 3D confocal microscope using blue light for both the conventional and the three-dimensional (DMTA) dental wear studies. All specimens were scanned in 3D and photos were taken from four adjacent areas at 20 × magnification (Fig. 1c, d). Each measured area was 637 × 468 μm (768 × 576 pixel). Surface elevations for each specimen were collected at a lateral (x, y) interval of 0.83 μm with a vertical resolution of 0.005 μm. Although higher magnification is most often used (e.g.Charles et al., 2007; Goillot et al., 2009; Mallon & Anderson, 2014; Rodrigues et al., 2009), this typically depends on the group being studied (the size of the tooth enamel area or the food size). In this case, higher magnification was not considered appropriate based on large tooth size. These scanned areas were converted to grayscale photosimulation bitmap images (Fig. 1c), which were used for 2D microwear analysis.

For easier interpretation, as it has not been quantified before, we also measured the area of the enlarged crushing tooth and the area of the cranium in the case of placodonts (Table 1). Area of the cranium and that of the largest palatine teeth were measured on the same specimen. Photographs of placodont specimens were used for the measurements from Rieppel (2001) which were analysed by ImageJ program. The number of specimens is very low due to the incompleteness of the fossil material. In *Cyamodus hildegardis* and *Paraplacodus broilii*, it was not possible to measure the area of the cranium, because they are highly deformed or incomplete. The exception is the in situ teeth within these cranium which could be measured.

**Table 1:** Area parameters of the cranium and the palatal enlarged tooth in placodonts

Species	Inventory number	Area of the cranium (mm^2^)	Area of the enlarged palatal tooth (mm^2^)
*Cyamodus rostratus*	UMO BT 748	9 450	420.9
*Cyamodus kuhnschnyderi*	SMN 15855	27 742	1 455.3
	SMN 16270	36 120	2 086.8
*Placodus gigas*	UMO BT uncatalogued	11 638	541.9
	UMO BT 13	18 358	577.2
*Psephoderma alpinum*	PIMUZ A/III 1491	8 577	434.3
	MSNM V471	5 823	277.4
*Henodus chelyops*	GPIT RE 7290 (II)	12 643	73
*Placochelys placodonta*	SZTFH Ob.2323, Vt.3	9 255	429.2
*Macroplacus raeticus*	BSP 1967 I 324	26 643	3 927.3

UMO = Urwelt–Museum Oberfranken, Bayreuth, Germany; SMN = Staatliches Museum für Naturkunde, Stuttgart, Germany; PIMUZ = Palaeontological Institute and Museum, University of Zurich, Switzerland; MSNM = Museo Civico di Storia Naturale di Milano, Italy; BSP = Bayerische Staatssammlung für Paläontologie und Historische Geologie München, Germany; SZTFH = the Department of Collections of the Supervisory Authority for Regulatory Affairs, formerly the Collection of the Hungarian Geological Institute, Budapest, Hungary; GPIT = Geologisch–Paläontologisches Institut, Universität Tübingen, Germany

### 2D microwear analysis

The microwear feature types that were analysed are scratches and pits. Pits have a width to length ratio higher than 1/4, while scratches have a lower one (Grine, 1986). The quantity and ratio of these features on the dental wear facet can be essential to separate the dietary categories or fine dietary differences between each species (Petraru et al., 2020; Rivals et al., 2022; Solounias & Hayek, 1993; Ungar, 1994). In total, six variables of these wear features were determined on 2D photosimulation images using the Microware 4.02 software: (1) number of pits, (2) number of scratches, (3) mean length of pits, (4) mean width of pits, (5) mean length of scratches, (6) mean width of scratches (Ungar, 2002). The counting of the wear features was done exclusively by K.G. Furthermore, the percentage of pits (pit% = 100 × Np/(Np + Ns) (Np = number of pits, Ns = number of scratches, e.g., Merceron & Ungar, 2005; Merceron et al., 2004) has been calculated to determine the proportion of pits.

### Dental microwear texture analysis (DMTA)

Each 3D point cloud was analysed by scale-sensitive fractal analysis (SSFA; Digital Surf Mountains 8) based on several previous studies (e.g.Caporale & Ungar, 2016; DeSantis et al., 2013; Merceron et al., 2014; Prideaux et al., 2009; Scott, 2012; Ungar et al., 2003, 2007). In the present study, the attributes of SSFA measured are the following: complexity, anisotropy, scale of maximum complexity and heterogeneity.

Complexity (area-scale fractal complexity = Asfc) shows the change in surface roughness at different scales. A surface dominated by pits of various sizes, or pits and scratches will tend toward high complexity. Animals consuming harder and/or more brittle food items, such as woody material, seeds, and fruit pits, have higher complexity (e. g. Scott, 2012; Scott et al., 2005). Anisotropy (exact-proportion length-scale anisotropy of relief = epLsar) is a measure of orientation concentration of surface roughness. If the features on the surface are arranged in a similar orientation (e.g., lots of parallel striations), the anisotropy is high. Scale of maximum complexity (Smc) measures the fine-scale limits of the Asfc, where the higher Smc values mean fewer small features. Heterogeneity of area-scale fractal complexity (HAsfc(9 × 9)) can be calculated by splitting of each adjacent area into subregions (9 × 9 grid, total of 81 subsamples). Surfaces with high heterogeneity have greater disparity in complexity values between subdivided samples and the entire surface.

### Statistical analysis

Data were statistically analysed with R Statistics Software (R Core Team, 2021). As multivariate analysis, the Principal Components Analysis (PCA) was used on the following variables:

- 2D microwear analysis: length of pits, width of pits, length of scratches and width of scratches
- 3D microwear analysis: complexity, anisotropy, scale of maximum complexity and heterogeneity.

The PCA was run separately for 2D and 3D analyses for placodonts, and 2D analysis also in association with mammals. The 3D analysis was not interpreted for mammals, because the 20 × magnification in their case was low and the original crown morphology of the tooth itself distorted the results. In contrast, in 2D analysis, the only observer (KG) could decide what counted as tooth wear feature.

The PCA calculates the component covering the largest proportion of variance (PC1), followed by the second component (PC2) that best explains the remaining variance, and so on. The components are orthogonal and can be treated as independent variables. The biplot of the first two components is used to visually display in the case of both methods. Furthermore, the number of microwear features and percentage of pits were compared between each species using bar plots and boxplots. Finally, the changes of two attributes of SSFA, the complexity and anisotropy were also examined. They can be used to show how varied the wear surface is, how uniform the size and depth of the wear is, whether the distribution is uniform or uneven. Additional file 1 contains the input data of 2D microwear analysis, while Additional file 2 includes the input data of 3D microwear analysis.

## Results

### 2D microwear analysis on fossils

**Fig. 2:**
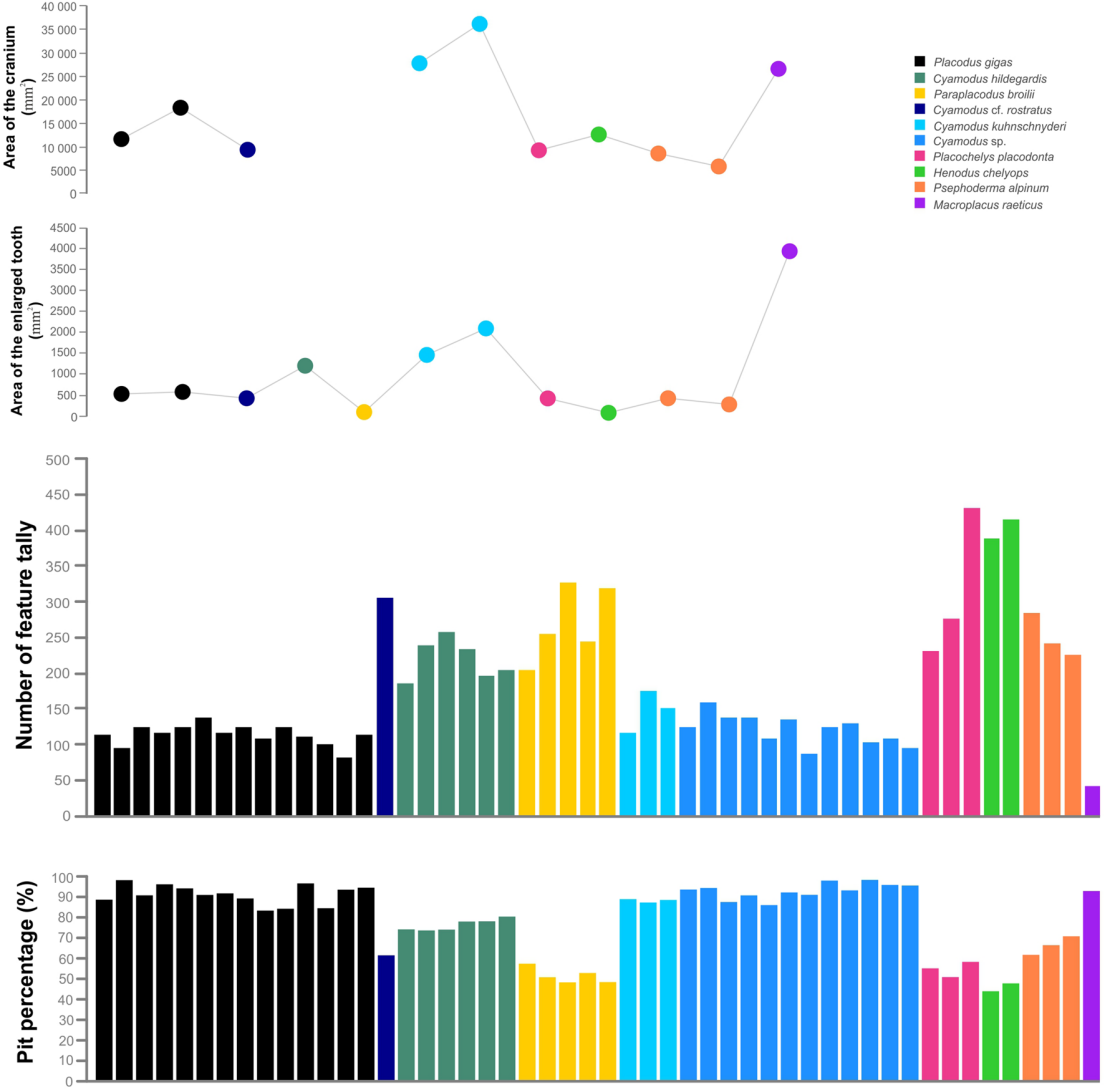
Results of the area of the cranium and the enlarged tooth; and the microwear feature number and the pit percentage plotted on a bar chart

The highest feature number (pits and scratches together) can be observed on *Henodus chelyops* (402 on average) and the holotype of *Placochelys placodonta* (432) (Table 2, Fig. 2). These are followed by *Cyamodus* cf. *rostratus* (307 on average), the referred material of *Placochelys placodonta* (231 and 276) and *Psephoderma alpinum* (251 on average). In the case of *Paraplacodus broilii* and *Cyamodus hildegardis* the range is similar (270 and 220, respectively), but the values are more scattered. A lower feature number can be detected on the other *Cyamodus* species: *Cyamodus kuhnschnyderi* has 147 features on average and the group of *Cyamodus* sp. has 121 features on average. The range of the *Placodus gigas* is similar to the latter group (between 82 and 138). *Macroplacus raeticus* shows the least wear feature number (42).

**Table 2:** Measured 2D microwear data

Species	N_s_	N_feature(µm)_		N_pit_		N_scratch_		Pit %		P_length(µm)_		P_width(µm)_		S_length(µm)_		S_width(µm)_	
		Mean	SD	Mean	SD	Mean	SD	Mean	SD	Mean	SD	Mean	SD	Mean	SD	Mean	SD
**Placodonts**																	
*Cyamodus hildegardis*	6	219.92	25.20	167.74	17.66	52.18	9.74	76.38	2.56	21.60	1.80	15.64	1.83	52.05	3.93	4.04	0.80
*Cyamodus* cfr. *rostratus*	1	307.00	-	188.75	-	118.25	-	61.48	-	20.95	-	16.31	-	53.03	-	4.99	-
*Cyamodus kuhnschnyderi*	3	146.67	24.52	129.25	20.69	17.42	3.88	88.23	0.70	26.49	2.67	20.43	2.63	53.35	3.69	4.21	0.53
*Cyamodus* sp.	12	120.78	19.91	112.26	18.41	8.52	4.67	93.03	3.64	25.13	2.33	20.55	2.71	96.80	48.11	3.27	1.60
*Placodus gigas*	14	113.89	13.55	103.73	12.58	10.16	5.35	91.18	4.58	27.12	1.67	21.93	1.97	86.22	43.29	3.86	2.11
*Psephoderma alpinum*	3	250.69	25.10	165.33	7.40	85.36	17.88	66.31	3.70	18.97	1.97	13.93	1.30	82.33	20.78	2.06	0.08
*Henodus chelyops*	2	402.67	13.67	185.00	14.00	217.67	0.33	45.88	1.92	13.48	0.61	9.82	0.87	73.59	3.97	1.49	0.03
*Placochelys placodonta*	3	313.44	86.09	173.42	55.83	140.03	31.39	54.76	3.04	19.11	2.18	14.59	2.09	86.97	9.54	1.81	0.12
*Paraplacodus broilii*	5	270.05	46.49	137.73	15.74	132.32	30.89	51.55	3.37	21.89	1.03	15.49	0.57	84.28	4.97	2.29	0.27
*Macroplacus raeticus*	1	42.00	-	39.00	-	3.00	-	92.86	-	46.94	-	38.19	-	32.39	-	2.17	-
**Extant mammals**																	
*Dugong dugon*	1	312.33	-	78.67	-	233.67	-	25.19	-	15.39	-	10.85	-	113.14	-	1.51	-
*Trichechus manatus*	1	306.33	-	118.33	-	188.00	-	38.63	-	12.91	-	8.83	-	118.11	-	1.77	-
*Enhydra lutris*	3	261.42	7.80	145.67	15.11	115.75	12.97	55.68	5.21	21.19	0.67	15.64	0.75	96.90	7.58	2.85	0.33

Abbreviations: N = number, Ns = number of specimen, P = pit, S = scratch, SD = standard deviation

Four taxa show similar high pit percentage values: *P. gigas* (91%), *M. raeticus* (93%), *C. kuhnschnyderi* (88%) and the group of *Cyamodus* sp. (93%) (Table 2, Fig. 2). These are followed by *C. hildegardis* with smaller value (76%). Similar pit percentage can be observed at *C.* cf. *rostratus* (61%), *P. broilii* (52%), *P. placodonta* (55%) and *P. alpinum* (66%), while *H. chelyops* has the lowest value (46%). Here, the scratches predominate opposed to pits. In addition, two of the *P. broilii* specimens show more scratches.

**Fig. 3:**
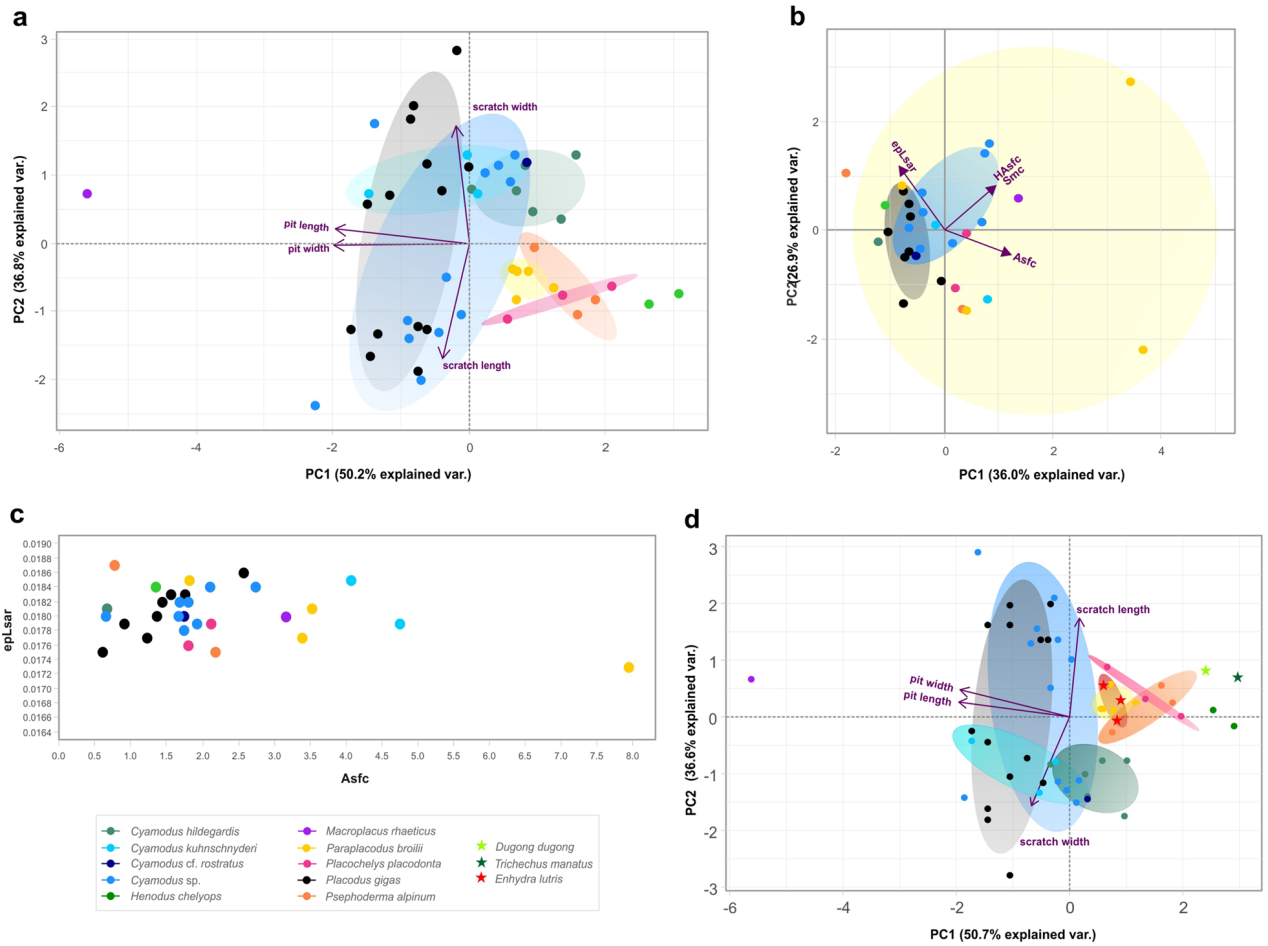
**a** Results of the 2D microwear analysis and (**b**) 3D microwear analysis (DMTA). In the case of the latter the PCA diagram based on the following variables: Asfc (complexity), epLsar (anisotropy), HAsfc (heterogeneity of area-scale fractal complexity), Smc (scale of maximum complexity). **c** Biplot showing anisotropy (epLsar) and complexity (Asfc). **d** results of the 2D microwear analysis based on placodonts and extant marine mammals

**Fig. 4:**
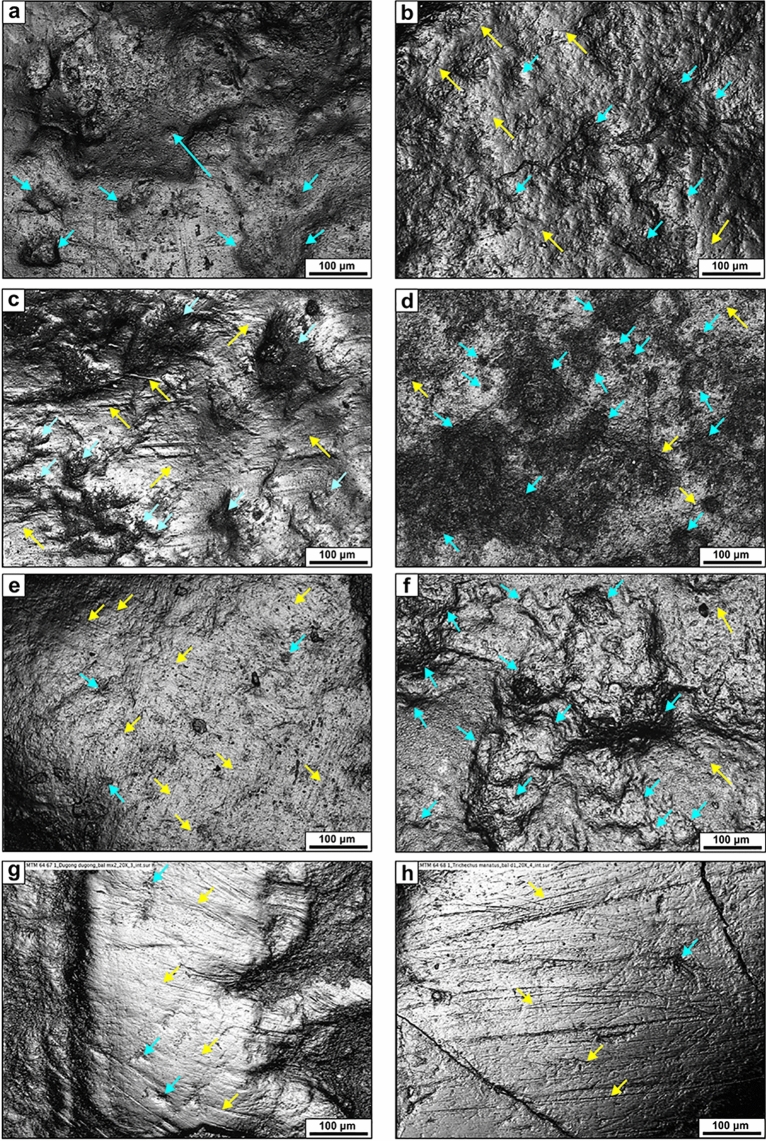
Photosimulations of the dental microwear facet of placodonts. The areas are 637 × 468 μm (= 20 × magnification). The light blue arrows show pits, while the yellow arrows indicate scratches. **a** BSP 1967 I 324 *Macroplacus raeticus*, left p1. **b** SZTFH Ob.2323, Vt.3 *Placochelys placodonta*, left p2 of holotype **c** PIMUZ T 5927 *Paraplacodus broilii* distal palatine tooth **d** SMF R 368 *Placodus gigas*, left d4 **e** GPIT/RE/7290 *Henodus chelyops* II., left d **f** SMNS 16270 *Cyamodus kuhnschnyderi*, left p3 **g** MTM 64.67.1 *Dugong dugon*, left mx2 **h** MTM 64.68.1 *Trichechus manatus*, left d1. Abbreviations: d = dentary tooth, p = palatal tooth, mx = maxillary tooth

The PC1 and the PC2 explain 87% of total variance, 50.2% and 36.8%, respectively (Table 3). The PC1 is explained by length and width of scratches, while the length and width of pits are stronger factors in the second component. The other two components (PC3 and PC4) are not exploited. *Macroplacus raeticus* is separated the most from the other species (Fig. 3a) having large pits (46.94 µm long and 38.19 µm wide on average) and relatively short, broad scratches (32.39 µm long and 2.17 µm wide on average) (Table 2, Fig. 4a). However, it is similar to *Placochelys placodonta*, *Paraplacodus broilii*, *Psephoderma alpinum* and to some specimens of *Cyamodus* sp. and *Placodus gigas* (Fig. 4b-–d). In contrast, the posterior crushing teeth of *H. chelyops* bear the smallest pits (13.48 µm long and 9.82 µm wide on average) together with long, narrow scratches (73.59 µm long and 1.49 µm wide on average) (Fig. 4e). *Paraplacodus broilii*, *P. alpinum* and *P. placodonta* are located near *H. chelyops* on the plot having similar parameters of scratches, but greater pit size. *Cyamodus hildegardis*, *C.* cf. *rostratus*, *C. kuhnschnyderi* have similar length and width of scratches, while the size of pits is different. *Cyamodus hildegardis* and *C*. cf. *rostratus* are characterised by smaller pits, while *C. kuhnschnyderi* show greater pits (26.49 µm long and 20.43 µm wide on average, Fig. 4f). The parameters of the group of *Cyamodus* sp. and *P. gigas* are scattered the most, when some of the specimens of these two groups bear short, wide scratches and others show long, narrow scratches. However, the size of the pits is similar in the case of *P. gigas*, while it covers a larger range in the specimens of *Cyamodus* sp.

**Table 3:** Results of PCA of 2D conventional microwear analysis

	PC1	PC2	PC3	PC4
Standard deviation	1.4172	1.2125	0.7119	0.12101
Proportion of variance	0.5021	0.3675	0.1267	0.00366
Cumulative proportion	0.5021	0.8696	0.9963	1
pit length	−0.6944	0.0871	0.1618	0.6957
pit width	−0.7025	−0.0144	0.0456	−0.7100
scratch length	−0.1404	−0.6980	−0.6937	0.1086
scratch width	−0.0671	0.7106	−0.7003	0.0070

### 3D microwear analysis on fossils

The two graphs show similar results (Fig. 3b, c). The largest variance in the DMTA variables can be observed in the specimens of *Paraplacodus broilii*, *Psephoderma alpinum* and *Cyamodus kuhnschnyderi*, where the values of complexity (Asfc) and anisotrophy (epLsar) can be both very high and low (Table 4). *Placodus gigas* and the group of *Cyamodus* sp., with the highest specimen number, cover a smaller range of values. *Henodus chelyops* has the highest epLsar (0.0184) and the lowest Asfc (1.339). It is followed by *Cyamodus hildegardis* and *Cyamodus* cf. *rostratus* with increasing Asfc (0.662 and 1.738), while the epLsar is smaller (0.018137 and 0.01796). The two specimens of *Placochelys placodonta* show smaller variance (Asfc = 1.9533, epLsar = 0.01772). *Macroplacus raeticus* is characterised by high Asfc (3.1547) and low epLsar (0.01797). In the case of the PCA, the first two components explain 63% of the total variance (Table 5). The PC1 is most strongly explained by complexity (Asfc), while in the case of PC2, anisotropy (epLsar) explains most of the variance. Thus, the multivariate and bivariate analysis show similarities. Well-separated groups cannot be observed in either case. The scale of maximum complexity and heterogeneity proved negligible in terms of multivariate analysis.

**Table 4:** Measured 3D microwear data

Species	N_s_	Asfc		Smfc (µm)		HAsfc9		epLsar	
		Mean	SD	Mean	SD	Mean	SD	Mean	SD
*Cyamodus hildegardis*	1	0.661580	-	11.781265	-	0.300884	-	0.018137	-
*Cyamodus* cfr. *rostratus*	1	1.738003	-	10.768998	-	0.372918	-	0.017962	-
*Cyamodus kuhnschnyderi*	2	4.405682	0.335175	8.820650	0.223123	0.366849	0.012021	0.018202	0.000280
*Cyamodus* sp.	8	1.781664	0.540714	13.843004	2.883546	0.680948	0.257188	0.018104	0.000207
*Placodus gigas*	8	1.419754	0.550038	10.683872	1.573018	0.405050	0.106599	0.018067	0.000347
*Psephoderma alpinum*	2	1.468178	0.703663	10.968535	1.397987	0.377327	0.021059	0.018107	0.000582
*Henodus chelyops*	1	1.338911	-	15.741954	-	0.363389	-	0.018426	-
*Placochelys placodonta*	2	1.953300	0.152884	9.765389	0.634149	0.589529	0.101891	0.017722	0.000130
*Paraplacodus broilii*	4	4.162421	2.280805	52.289384	68.840136	0.554040	0.144279	0.017930	0.000446
*Macroplacus raeticus*	1	3.154681	-	15.402506	-	0.954394	-	0.017974	-

Abbreviations: N_s_ = number of specimen, Asfc = complexity, Smc = scale of maximum complexity, HAsfc9 = heterogeneity of area-scale fractal complexity, epLsar = anisotropy, SD = standard deviation

**Table 5:** Results of PCA of 3D microwear analysis

	**PC1**	**PC2**	**PC3**	**PC4**
Standard deviation	1.2004	1.0367	0.8982	0.8232
Proportion of variance	0.3602	0.2687	0.2017	0.1694
Cumulative proportion	0.3602	0.6289	0.8306	1.0000
Asfc	0.6198	−0.2616	0.1254	0.7292
epLsar	−0.4120	0.6940	0.1104	0.5801
HAsfc	0.4611	0.4715	−0.7457	−0.0945
Smc	0.4832	0.4772	0.6449	−0.3504

### Analysis of the extant marine mammals

*Dugong dugon* is characterised by the highest feature number (312), but the value of *Trichechus manatus* is equally high (306) (Table 3). However, *Enhydra lutris* shows an average of 261 wear feature number. Percentage of pits is similarly low in *D. dugon* and *T. manatus* (25% and 39%, respectively), while in *E. lutris* it is slightly more than 50%.

PCA of the 2D features together with placodonts is very similar to the results of the 2D analysis on just placodonts (Fig. 3d). The PC1 and the PC2 explain 87% of total variance, 50.7% and 36.6%, respectively (Table 6). The PC1 is also affected by length and width of scratches, while the length and width of pits and length of scratches are stronger factors in the second component. *Dugong dugon* and *Trichechus manatus* move together with *Henodus chelyops*, having the smallest pits (15.39 µm long and 10.85 µm wide in *Dugong*; 12.91 µm long and 8.83 µm wide in *Trichechus*) (Table 2, Fig. 4g, h). The scratches are narrow and long (113 µm long in *Dugong* and 118 µm long in *Trichechus*).

**Table 6:** Results of PCA of 2D microwear analysis (fossils and extant mammals)

	**PC1**	**PC2**	**PC3**	**PC4**
Standard deviation	1.4244	1.2100	0.7023	0.11650
Proportion of variance	0.5073	0.3660	0.1233	0.00339
Cumulative proportion	0.5073	0.8733	0.9966	1
pit length	−0.69059	0.10336	−0.14258	0.70149
pit width	−0.67917	0.19632	−0.04167	−0.70601
scratch length	0.06555	0.72098	0.68298	0.09711
scratch width	−0.23985	−0.65648	0.71517	0.00597

*Enhydra lutris* has larger pits and slightly shorter and wider scratches than the other two mammals. Pits are 21.19 µm long and 15.64 µm wide, while scratches are 96.9 µm long and 2.85 µm wide on average. It is closest to the placodonts *Paraplacodus broilii*, *Placochelys placodonta* and *Psephoderma alpinum*.

## Discussion

### Comparison of tooth microwear patterns in placodont species

Traditional microwear analysis are mainly used on terrestrial herbivorous mammals, such as ungulates (Merceron & Ungar, 2005; Merceron et al., 2004; Solounias & Semprebon, 2002) and primates (Ungar et al., 2008), but there are only a few studies on carnivore mammals (Goillot et al., 2009), crocodiles (Ősi & Weishampel, 2009), dinosaurs (Mallon & Anderson, 2014; Whitlock, 2011) and lizards (Gere et al., 2021). Comparison of these analyses to those of placodonts, however, is complicated. Placodonts lived in marine environments (maybe with exception of *Henodus chelyops* which was suggested that lived in playa lake or lagoonal–brackish environment ecology; Reif, 1937; Pinna, 1990), where various factors influencing tooth wear process in a terrestrial environment (such as grit, dust, phytoliths; Solounias & Semprebon, 2002) did not exist. On the other hand, the previous results of the morphological observations (skull and tooth morphology, change in size, shape and number of teeth) suggest that the placodonts could have been dominantly durophagous consuming hard-shell food, such as bivalves, gastropods, brachiopods, and echinoids (e.g.Neenan, 2014; Rieppel, 2001, 2002). Thus, they are primarily compared to terrestrial groups that may have consumed hard/brittle objects, such as seed eaters, frugivores and bone eaters in mammals, or molluscivores in lizards. These groups are characterised by the dominance of pits (Gere et al., 2021; Goillot et al., 2009; Ungar et al., 2006), higher complexity than herbivores and higher roughness parameters (Purnell & Darras, 2016; Winkler et al., 2019).

The 2D dental microwear analysis supports the morphological observations, suggesting that the majority of placodont taxa may have been real durophages. However, our results clearly show that the nine placodont species studied are characterised by somewhat different wear patterns, presumably due to the somewhat different diet composition.

**Fig. 5:**
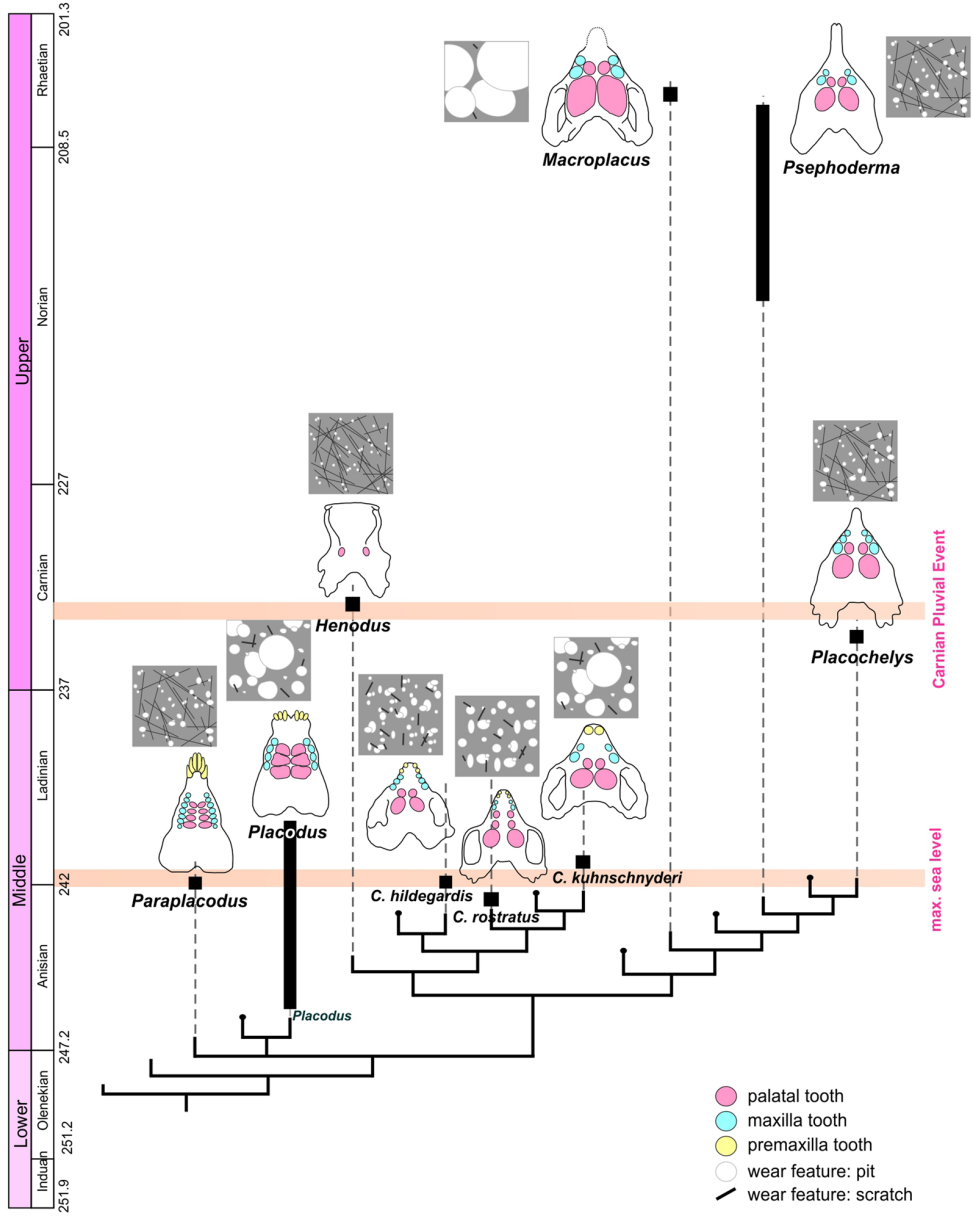
Evolutionary development of Placodontia based on morphology and dental microwear pattern. (modified after Wang et al. (2019))

**(1) Teeth of the early placodonts: *Paraplacodus broilii*–*Placodus gigas***: They represent the non-cyamodontoid placodonts and are the earliest-branching placodonts with skulls showing numerous plesiomorphic features (Neenan et al., 2015), and both have a relatively high number of teeth (Fig. 5): in *P. gigas*, there are three pairs of premaxillary, four pairs of maxillary teeth, and three pairs of palatal teeth, whereas *P. broilii* have three pairs of premaxillary, at least seven pairs of maxillary and four pairs of palatal teeth. *Placodus gigas* has much larger teeth than *P. broilii* relative to skull size, while the teeth of *P. broilii* had more complex morphology (Gere at el., 2020). Consistent with this, dental microwear pattern shows significant differences between the two species. *Placodus gigas* is characterised by a low number of wear features with a high percentage of pits, while wear surfaces of *P. broilii* show a high feature number with a lower pit percentage. Variations in the size of individual wear features are also observed. *Paraplacodus broilii* has long, narrow scratches and small pits. In contrast, *P. gigas* shows large pits with relatively variable scratch sizes.

It should be noted that the fossils of *P. gigas* used in this study are from the early Anisian to the Ladinian (e.g.Drevermann, 1933; Rieppel, 2001; von Huene, 1946; Westphal, 1967, 1988), while *P. broilii* remains have been found exclusively from the Anisian–Ladinian boundary, although the origin of the species itself can also be traced back to the early Anisian (e.g.Peyer, 1931b; Rieppel, 2000). There is also an important habitat difference between the two species. *Placodus gigas* is only known in the Germanic Triassic, representing an epicontinental marine environment (Mazin & Pinna, 1993), whereas *P. broilii* is known from the Alpine Triassic (Monte San Giorgio) in a carbonate platform marine environment (Kuhn-Schnyder, 1974; Peyer, 1931a, 1931b; Pinna, 1992).

**(2) Cyamodontidae**: This group includes *Cyamodus* cf. *rostratus* and *Cyamodus hildegardis* from the Anisian–Ladinian boundary, and *Cyamodus kuhnschnyderi* and the group of *Cyamodus* sp. from the Ladinian as the last cyamodontid in the western Tethys. When comparing them from the Anisian–Ladinian boundary to the end of the Ladinian, a clear trend in both cranial and dental morphology and dental wear pattern can be observed. Skull morphological trends within the taxon can be identified in that the rostrum has become shorter, and the temporal region has widened during their evolution (Fig. 5). The late Anisian *C. rostratus* had an even longer rostrum and a narrow temporal region, whereas the Ladinian *C. kuhnschnyderi* had a short rostrum and a strongly widened temporal region. *Cyamodus hildegardis*, on the other hand, shows an intermediate state at the Anisian–Ladinian boundary. The dentition follows this evolutionary change: decreasing number of teeth and increasing tooth size (Fig. 2). *Cyamodus rostratus* had two pairs of premaxillary and maxillary teeth and three pairs of palatal teeth, while *C. kuhnschnyderi* had two pairs of premaxillary, maxillary and palatal teeth. *Cyamodus hildegardis* is also a transitional form. The results of the microwear analysis confirm this intra-group trend. The number of wear features showed a decrease, while the percentage of pits increased during the evolution. The scratches have the same length and width (short and wide), but an increase in size of pits can be observed. These results suggest that larger, harder-shelled food increasingly became more dominant during the Anisian–Ladinian evolutionary history of the genus *Cyamodus*.

Compared to the non-cyamodontoid forms *Paraplacodus broilii* and *Placodus gigas*, *Cyamodus* has a wider temporal region, an elongated rostrum, decreasing number of teeth, and teeth became less complex and larger. *Cyamodus* cf. *rostratus* and *Cyamodus hildegardis* are similar to *Paraplacodus,* because they have high wear feature number and small pits, but *Paraplacodus* shows lower pit percentage and narrower scratches. The Ladinian cyamodontids show similarities with *Placodus*, having low feature number, high pit percentage and large pits.

What *Cyamodus* cf. *rostratus* and *Cyamodus hildegardis* have in common is a similar appearance around the Anisian–Ladinian boundary, although the former appears slightly earlier in the fossil record, i.e., in the Illyrian (von Meyer, 1855; Rieppel, 2001). At this time, there was a major sea-level rise (Kelley et al., 2014). *Paraplacodus broilii* and *Cyamodus hildegardis* are only known from the Alpine Triassic at Monte San Giorgio (carbonate platform marine environment) (Kuhn-Schnyder, 1974; Peyer, 1931a, 1931b; Pinna, 1992), whereas *Cyamodus* cf. *rostratus* is from the Germanic Triassic (epicontinental marine environment). The differences and similarities in microwear pattern may be due to the difference in environment, the rise in sea level at that time and the related food supply. *Cyamodus kuhnschnyderi* and the group of *Cyamodus* sp. are from similar environment (Germanic Triassic) (Gere et al., 2020; Hagdorn & Rieppel, 1998; Nosotti & Pinna, 1996), and at this geological time, in Ladinian, there was a sea-level fall and a gradual reduction of the epicontinental marine areas (Pinna, 1990).

**(3)** ***Placochelys placodonta–Psephoderma alpinum***: The skulls and dentition of the two species are very similar and both are members of Placochelyidae (Neenan et al., 2015; Wang et al., 2020). However, they differ in age, because *Placochelys placodonta* is from the Carnian, while *Psephoderma alpinum* fossils are known from the middle Norian to the middle of the Rhaetian (Neenan et al., 2013; Wang et al., 2019). They have a very specific cranial morphology compared to earlier forms (*Placodus*, *Paraplacodus*, *Cyamodus*). They are characterised by a wide temporal region, such as the *Cyamodus*, but the rostrum is strongly elongated and completely toothless (Fig. 5). Their maxillary and palatal teeth are similar to *Cyamodus* in number but smaller in size. As with the general morphology, the dental microwear patterns of *Placochelys placodonta* and *Pspehoderma alpinum* are very similar. They show a high number of wear features and low pit percentage, such as *Paraplacodus broilii* and *Cyamodus hildegardis*. The size parameters of individual wear features are also similar to *Paraplacodus,* having small pits and long, narrow scratches.

**(4) *Henodus*, an alternative form**: The phylogenetic position of *Henodus* is even more highly nested within Placodontia (Wang et al., 2022) with specialised jaw and dental morphology (only one pair of posterior, concave crushing teeth; anterior denticles with flat, spatula-like rostrum; potential baleen-like lamellar structures in the jaws: Rieppel, 2001; von Huene, 1936). Compared to other placodonts, it inhabited playa lake environment (Pommery et al., 2021; Reif & Stein, 1999; Rieppel, 2001, 2002) and its ecology is that of a lagoonal–brackish species (Hagdorn & Rieppel, 1998; Pinna, 1990; Reif, 1937). It has a very high number of wear features and the pit percentage is 46% on average, which is unique to this species. The size of the pits is very small compared to the earlier species (Ladinian forms and *Placochelys*), and the scratches are very long and narrow. *Henodus chelyops* has the longest scratches and the smallest pits among placodonts.

*Henodus* is of similar age to *Placochelys,* but the latter lived in shallow marine environments (Csillag & Haas, 1993). The temporal occurrence of both species can be placed close to the Carnian Pluvial Event. This event is characterised by increased precipitation, and hence increased transport of terrigenous material into shallow sea regions (Ogg, 2015). It resulted in changes in the fauna, with some groups disappearing and others appearing (Reif, 1937). This may also have affected the environment of these placodonts, with a potential change of their diet composition. In the case of *H. chelyops*, the high number of scratches supports the hypothesis that it consumed not only hard-shelled food but its diet could be mixed, e.g., plant material or *Estheria* crustaceans (Reif & Stein, 1999). In Pommery et al. (2021), specifically also the small gastropods found with the *Henodus* skull were discussed as a potential food source. Recently, another skull assignable to the genus *Henodus*, was described from Rocha da Pena site, Algarve region, Portugal in a Master thesis (Ruciński, 2020). The find represents the first skull assignable to this genus outside of southwestern Germany. The study further mentions the coexisting faunal components, consisting of isolated and scattered remains of bony fishes, hybodontiform sharks, and reptile postcranial bones, potentially including other cyamodontoid placodonts. Ruciński (2020) noted also the absence of invertebrate remains from these layers the skull was found in, but indicated that this lack could be caused by diagenetic alteration of the sediments at the Rocha da Pena site.

**(5) The placodont with the largest enlarged palatal teeth, *Macroplacus raeticus***: The only known specimen came from Germany (Bavarian Alps—Allgäu) (Schubert-Klempnauer, 1975). Its skull is similar in size to that of the Ladinian *Cyamodus*. Very prominent in this taxon are the extremely enlarged distal, rhomboid-shaped palatal teeth. Compared to the size of the skull, this species has the largest posterior crushing teeth among placodonts (Fig. 5). Dental microwear pattern is also markedly different from the others. It has the lowest number of wear features, but almost 100% of the worn surfaces have only pits, which are the largest in size (in contrast to the other taxa). Enlarged teeth and the large individual wear features may indicate larger hard-shelled food consumption.

The results of 3D dental wear analysis (DMTA) cannot be included in the above discussion, because data are difficult to interpret. Anisotropy values fall within a narrow range for all specimens (0.0173–0.0187), with no distinct groups. Even values for individuals within a species are highly scattered. Orientation cannot be observed supporting the fact that special jaw movement or chewing mechanism could not have existed in placodonts (Rieppel, 2002) or, in the case of *Henodus chelyops*, was still quite limited (Pommery et al., 2021). The complexity results also show no significant differences, but some increasing trend can be demonstrated: *Cyamodus hildegardis* (0.6616); *H. chelyops* (1.3389); *Placodu*s *gigas* (0.5963–2.5633, average 1.4198); *Cyamodus* cf. *rostratus* (1.7380), *Cyamodus* sp. (0.6435–2.7297, average 1.7817), and *Placochelys placodonta* (1.8004–2.1062); *Macroplacus raeticus* (3.1547); *Cyamodus kuhnschnyderi* (4.0705–4.7409). However, no similar correlation to the results of the 2D tooth wear analysis was observed.

Overall, significant differences cannot be detected. The results of 3D analysis should be handled with caution. Although the most intact enamel has been selected, it was not possible to completely avoid cracks and other traces of dissolution to affect the results. However, it should also be noted that these fossils are very old and the most of which were obtained from calcareous rock. Researchers started using 3D method for young fossils and extant mammals but there are some publications on dinosaurs now. It is important that the enamel have to be intact, because the entire surface of the tooth enamel is scanned, including cracks and other damages. Thus, the results will be affected.

Assuming this information, basically, two groups can be distinguished in the results of dental wear patterns (based on 2D conventional microwear analysis): (1) the first group has high feature number (well over 200, even over 400) and relatively lower percentage of pits (between 46% and 76%), including *Cyamodus* cf. *rostratus*, *C. hildegardis*, *Paraplacodus broilii*, *Placochelys placodonta*, *Henodus chelyops* and *Psephoderma alpinum*; (2) the second group has lower feature number (under 150) and very high percentage of pits (around 90%), including *Placodus gigas*, *C. kuhnschnyderi*, the group of *Cyamodus* sp. and *Macroplacus raeticus*.

The wear pattern is highly correlated with the number and size of teeth (Fig. 2). It may even be related to the size of the food. Species with larger teeth were more able to crush larger (possibly harder-shelled) food, which could cause less, but larger individual wear features. In contrast, species with smaller teeth might have processed smaller (possibly thinner-shelled) food efficiently, which could result in more, but smaller individual wear features. The results of microwear analysis suggest that there may have been a change in the composition of the hard-shelled invertebrate fauna, especially in their size or in other shell characteristics (ornamentation, shell thickness). A few observations has been shown that the crushing of molluscs and gastropods may require different forces, and the structure or material of the shell (fibrous or prismatic microstructure, aragonite or calcite) can be important factors (Kolmann et al., 2015). In the future, the fossil hard-shelled invertebrate fauna should be examined to see there have been any changes during the Triassic and then, it can be compared with the changes observed in placodonts. For example, in brachiopods there was an increase in diversity around the Anisian–Ladinian boundary, followed by a decrease in the Ladinian and then a repeated increase in the Carnian and Norian when they reached their maximum diversity (Kocsis, 2015). The higher diversity phases are correlated to the first group of microwear patterns (*Cyamodus* cf. *rostratus*, *C. hildegardis*, *Paraplacodus broilii*, *Placochelys placodonta*, *Henodus chelyops* and *Psephoderma alpinum*) having high feature number but relatively low pit percentage. This suggests that although the diversity of hard-shelled species increased, there were presumably many additional food sources available, resulting in a finer and more scratch-rich wear surface in various placodont species.

#### Comparison of tooth microwear patterns between placodonts and extant marine mammals

The number of wear feature is high for both extant marine mammal dugong (*Dugong dugon*) and manatee (*Trichechus manatus*) (Table 2). Looking in more details at the distribution of pits and scratches, they have very low pit percentages, meaning that the dominance of scratches is significant. They have the smallest pits, associated with long and narrow scratches. All results considered, *Henodus chelyops* is the most similar to them. The mammal sea otter (*Enhydra lutris*) shows similarities with the placodont *Psephoderma alpinum, Placochelys placodonta* and *Paraplacodus broilii*. The other placodonts (*Cyamodus kuhnschnyderi*, *Placodus*, *Macroplacus*) are different showing low number of wear feature, high pit percentage, large pits. *Cyamodus hildegardis* and *Cyamodus rostratus* are similar to sea otters in the number of wear feature and the percentage of pits.

Based on the above, the diet of *Henodus chelyops* may show similarities to dugong and manatee. The dugong feeds mainly on seagrasses, but also on small invertebrates (polychaete) that have settled on the plant (Lanyon & Sanson, 2006; Preen, 1995). The diet of manatee is also largely composed of seagrass, but it also consumes freshwater grasses, algae (Castelblanco-Martínez et al., 2009) and, in some cases, invertebrates (Courbis & Worthy, 2003). This supports the hypothesis that *Henodus* was no longer predominantly a hard-shelled prey feeder, but may have included plants in its diet (Pommery et al., 2021). There is also a similarity with herbivorous (*Iguana iguana*) and algaevorous lizards (*Amblyrhynchus cristatus*), where many narrow scratches can be observed (Winkler et al., 2019). It should be borne in mind that the composition of the marine vegetation was not quite the same at that time. Ancestor of sea grasses only appeared 100 million years ago (Hemminga & Duarte, 2000). Fossil plant remains are scarce and not well preserved, so we do not know their exact composition. However, some plants and even algae remains have been identified in El Atance fossil site, where a partial skull of the henodontid placodont *Parahenodus* was found (García-Ávila et al., 2021). Values of *Placochelys placodonta*, *Psephoderma alpinum* and *Paraplacodus broilii* are most comparable to the sea otter, which feeds mainly on clams, sea urchin, crabs and mussels (Kvitek et al., 1993; Maldini et al., 2010), very rarely fish and fish eggs (Lee et al., 2009). Values of other placodonts, such as *Placodus gigas*, *Cyamodus* sp. and *Macroplacus raeticus*, differ from these three extant marine mammals. Previous research has shown that they are similar to frugivorous mammals (Solounias & Semprebon, 2002; Solounias et al., 1988) and reptiles that eat hard-shelled food (Gere et al., 2021). This similarity may be explained because of the high pit dominance. Although sea otters also eat hard-shelled food and a similar tooth wear pattern would be expected for *Placodus gigas*, *Cyamodus* sp. and *Macroplacus raeticus*, but still show a difference. These placodonts are characterised by low number of wear feature, high pit percentage and much larger pits. This could possibly be because the food may have been larger than that consumed by sea otters or *Placochelys placodonta*, *Psephoderma alpinum* and *Paraplacodus broilii*. The larger food could have left larger tracks on the occlusal tooth surface.

## Conclusions

Many adaptations in skull morphology and dentition suggest that placodonts are one of the most specialized durophages among the Triassic marine reptiles. The 2D and 3D tooth wear analyses of placodont reptiles have clearly shown that even in these durophagous marine forms, different wear patterns due to somewhat different food consumption and prey/food item preference can be clearly detected. Our results revealed that 2D microscopy values correlate mostly with the number and size of the crushing teeth. Taxa having large distal palatine/dentary teeth (relative to the whole size of the skull, i.e., *Placodus gigas*, *Cyamodus* species, *Macroplacus raeticus*) show much fewer wear features and much larger pits than forms with smaller teeth (*Paraplacodus broilii*, *Placochelys placodonta*, *Psephoderma alpinum*, *Henodus chelyops*). Likewise, these two groups are separated in the number and size parameters of the wear features. However, DMTA data are more dispersive and show no discernible correlation with changes in dentition (number and size of tooth). Two morphologically extreme species stand out markedly from the others. *Macroplacus raeticus* with the largest, specific rhombus-shaped crushing teeth shows the fewest wear features (almost 100% pits) and the largest pits, while *Henodus chelyops* having the most reduced dentition is characterised by high wear feature number, the smallest pits and long, narrow scratches. *Henodus chelyops* is specialised among placodonts in terms of both skull shape and dentition to suggest that its diet and feeding mode could be more complex. This is confirmed by the microwear analysis, because *H. chelyops* is characterised by more scratches than pits. Based on observations in recent marine mammals (*Dugong dugon*, *Trichechus manatus*), its diet may not only consist of hard-shelled prey items (*Estheria* crustaceans), but also possibly plant material, such as algae. There may also be similarities with algaevorous (*Amblyrhynchus cristatus*) and herbivorous (*Iguana iguana*) lizards.

The different morphological adaptations in placodonts could be caused by the quality of the food, such as shell thickness and size, ornamentation and shape, as well as the composition of the shell (calcite, aragonite). These parameters can all influence the wear pattern, although the most important ones are size and the shell thickness of the food. It should also be noted that there are differences between Alpine and Germanic Triassic placodonts in the pattern of tooth wear which could be explained by taxic differences of the invertebrate fauna of the two provinces. In the future, comparing the invertebrate record (bivalves, gastropods, brachiopods, crustaceans, echinoids etc.) of the two areas may provide much useful information to better understand placodont food preferences.

### Supplementary Information

**Additional file 1.** Results of 2D microwear analysis for all specimens.

**Additional file 2.** Results of 3D microwear analysis for all specimens.

## Data Availability

The data sets supporting the conclusions of this article are included within the article and its additional files.
