# Supplementary material for: Complex dental wear analysis reveals dietary shift in Triassic placodonts (Sauropsida, Sauropterygia)
Source: Swiss J Palaeontol. 2024 Feb 5;143(1):4. doi: 10.1186/s13358-024-00304-x (PMC10844150; doi:10.1186/s13358-024-00304-x)
Supplement: Supplementary file 1 — Additional file 1. Results of 2D microwear analysis for all specimens. [file 13358_2024_304_MOESM1_ESM.docx]

**Supplementary 1** Results of 2D microwear analysis for all specimens. Abbreviations: N=number, P=pit, S=scratch, SD=standard deviation, d=dentary tooth, mx=maxilla tooth, p=palatine tooth.

| **Taxa** | **Inventory number** | **Element/tooth** | **N_feature_** | | **N_pit_** | | | | **N_scratch_** | | | | | **Pit %** | **P_length_ _(µm)_** | | | | | **P_width_ _(µm)_** | | | | | **S_length_ _(µm)_** | | | | | | **S_width_ _(µm)_** | | | |
| --- | --- | --- | --- | --- | --- | --- | --- | --- | --- | --- | --- | --- | --- | --- | --- | --- | --- | --- | --- | --- | --- | --- | --- | --- | --- | --- | --- | --- | --- | --- | --- | --- | --- | --- |
|  |  |  | mean | SD | mean | | | SD | mean | | | | SD |  | mean | | | | SD | mean | | | | SD | mean | | | | SD | | mean | | | SD |
| *Cyamodus hildegardis* | PIMUZ T 4763 | right d4 | 185.75 | 27.86 | 137.75 | | | 23.38 | 48.00 | | | | 6.60 | 74.16 | 24.92 | | | | 2.22 | 18.38 | | | | 2.11 | 58.50 | | | | 4.10 | | 4.17 | | | 0.74 |
|  | PIMUZ T 4770 | right p2 | 240.00 | 35.02 | 176.75 | | | 24.53 | 63.25 | | | | 13.99 | 73.65 | 22.21 | | | | 0.76 | 15.30 | | | | 0.74 | 52.13 | | | | 2.37 | | 4.79 | | | 0.13 |
|  | PIMUZ T 4771 | right p3 | 256.67 | 29.41 | 190.00 | | | 17.11 | 66.67 | | | | 15.54 | 74.03 | 19.60 | | | | 0.98 | 12.59 | | | | 0.21 | 51.41 | | | | 1.94 | | 5.24 | | | 0.35 |
|  | PIMUZ T 2796 | left p2 | 233.75 | 13.81 | 182.25 | | | 9.44 | 51.50 | | | | 6.10 | 77.97 | 19.66 | | | | 0.57 | 14.48 | | | | 0.65 | 54.91 | | | | 3.27 | | 3.20 | | | 0.43 |
|  | PIMUZ T 4768 | right p2 | 197.67 | 54.02 | 154.33 | | | 43.05 | 43.33 | | | | 11.03 | 78.08 | 22.01 | | | | 1.74 | 16.86 | | | | 1.86 | 49.09 | | | | 4.19 | | 3.82 | | | 0.30 |
|  | PIMUZ T 58 | right d2 | 205.67 | 18.70 | 165.33 | | | 15.80 | 40.33 | | | | 4.71 | 80.39 | 21.18 | | | | 1.39 | 16.23 | | | | 1.19 | 46.27 | | | | 1.10 | | 3.02 | | | 0.17 |
| *Cyamodus* cfr. *rostratus* | SMNS 17403 | left p2 | 307.00 | 48.85 | 188.75 | | | 21.59 | 118.25 | | | | 28.86 | 61.48 | 20.95 | | | | 0.82 | 16.31 | | | | 0.58 | 53.03 | | | | 3.75 | | 4.99 | | | 0.82 |
| *Cyamodus kuhnschnyderi* | SMNS 15855 | right p2 | 115.00 | 11.29 | 102.25 | | | 11.03 | 12.75 | | | | 3.90 | 88.91 | 30.16 | | | | 1.76 | 24.13 | | | | 1.75 | 53.55 | | | | 4.47 | | 3.67 | | | 0.48 |
|  | SMNS 16270 | left p3 | 174.75 | 18.99 | 152.50 | | | 16.26 | 22.25 | | | | 4.26 | 87.27 | 25.43 | | | | 1.68 | 18.36 | | | | 2.20 | 48.74 | | | | 6.20 | | 4.94 | | | 0.73 |
|  | SMNS 18380 | left d2 | 150.25 | 10.35 | 133.00 | | | 5.52 | 17.25 | | | | 5.12 | 88.52 | 23.87 | | | | 0.82 | 18.79 | | | | 0.99 | 57.76 | | | | 4.97 | | 4.02 | | | 0.53 |
| *Cyamodus* sp. * | SMNS 15990 | isolated | 125.00 | 8.40 | 117.00 | | | 7.71 | 8.00 | | | | 1.22 | 93.60 | 23.40 | | | | 1.33 | 17.61 | | | | 1.34 | 35.79 | | | | 4.02 | | 4.05 | | | 1.04 |
|  | SMNS 81181 | isolated | 159.67 | 31.63 | 150.67 | | | 28.86 | 9.00 | | | | 4.97 | 94.36 | 22.94 | | | | 2.20 | 17.07 | | | | 2.35 | 34.58 | | | | 2.28 | | 3.45 | | | 0.63 |
|  | SMNS 91472 | left p2 | 138.67 | 3.09 | 121.33 | | | 1.89 | 17.33 | | | | 4.50 | 87.50 | 23.82 | | | | 1.10 | 18.44 | | | | 1.19 | 40.30 | | | | 2.57 | | 3.99 | | | 0.53 |
|  | SMNS uncatalogued | isolated | 137.00 | 15.51 | 124.33 | | | 14.38 | 12.67 | | | | 4.92 | 90.75 | 22.43 | | | | 1.22 | 16.79 | | | | 0.83 | 33.20 | | | | 1.49 | | 4.33 | | | 0.55 |
|  | SMF R 5030 | isolated | 109.00 | 1.87 | 93.75 | | | 13.39 | 15.25 | | | | 11.84 | 86.01 | 26.31 | | | | 0.39 | 22.06 | | | | 0.47 | 131.69 | | | | 26.65 | | 2.83 | | | 0.49 |
|  | SMF R 5037 | isolated | 134.25 | 6.53 | 123.75 | | | 7.26 | 10.50 | | | | 2.50 | 92.18 | 25.80 | | | | 0.41 | 22.17 | | | | 0.67 | 140.11 | | | | 19.41 | | 2.57 | | | 0.31 |
|  | SMF R 4040 | left d4 | 86.25 | 4.49 | 78.50 | | | 3.57 | 7.75 | | | | 1.92 | 91.01 | 31.15 | | | | 1.37 | 26.27 | | | | 1.34 | 171.20 | | | | 24.89 | | 1.42 | | | 0.21 |
|  | SMF R 4039 | left d2 | 123.00 | 6.52 | 120.50 | | | 5.41 | 2.50 | | | | 1.80 | 97.97 | 25.38 | | | | 1.09 | 21.60 | | | | 0.97 | 144.60 | | | | 84.39 | | 1.38 | | | 0.81 |
|  | SMF R 5028 | isolated | 128.50 | 7.63 | 119.75 | | | 7.89 | 8.75 | | | | 1.79 | 93.19 | 24.43 | | | | 0.81 | 20.51 | | | | 0.77 | 136.95 | | | | 11.59 | | 2.71 | | | 1.07 |
|  | SMNS 56506 | isolated | 103.75 | 4.26 | 102.00 | | | 5.24 | 1.75 | | | | 1.48 | 98.31 | 27.60 | | | | 0.86 | 23.67 | | | | 0.36 | 87.84 | | | | 61.94 | | 7.65 | | | 11.04 |
|  | MTM VER 2013.15. | isolated | 108.50 | 9.21 | 104.00 | | | 9.03 | 4.50 | | | | 1.12 | 95.85 | 23.31 | | | | 0.32 | 19.92 | | | | 0.22 | 114.35 | | | | 25.07 | | 2.35 | | | 0.65 |
|  | MTM VER 2019.15. | isolated | 95.75 | 6.14 | 91.50 | | | 4.15 | 4.25 | | | | 3.34 | 95.56 | 24.94 | | | | 1.06 | 20.46 | | | | 1.03 | 90.95 | | | | 54.96 | | 2.57 | | | 1.61 |
| *Placodus gigas* | SZTFH V.15164 | d3 | 114.50 | 16.38 | 101.50 | | | 11.15 | 13.00 | | | | 6.40 | 88.65 | 29.20 | | | | 2.05 | 23.14 | | | | 1.81 | 48.95 | | | | 3.81 | | 3.41 | | | 0.29 |
|  | PIMUZ A/III0871 | left p2 | 96.00 | 16.36 | 94.25 | | | 14.58 | 1.75 | | | | 2.49 | 98.18 | 24.60 | | | | 0.73 | 19.10 | | | | 0.89 | 48.33 | | | | 63.25 | | 8.54 | | | 11.68 |
|  | SMF R 368 | left d4 | 124.50 | 6.54 | 113.00 | | | 8.03 | 11.50 | | | | 3.91 | 90.76 | 30.10 | | | | 1.13 | 24.52 | | | | 1.26 | 53.64 | | | | 9.17 | | 3.28 | | | 0.41 |
|  | SMF R 361 | left p1 | 116.75 | 12.74 | 112.25 | | | 11.10 | 4.50 | | | | 3.35 | 96.15 | 26.28 | | | | 1.67 | 20.99 | | | | 1.77 | 36.73 | | | | 21.39 | | 3.13 | | | 1.87 |
|  | SMF R 362 | left p2 | 124.00 | 7.48 | 116.75 | | | 6.76 | 7.25 | | | 4.21 | | 94.15 | 26.65 | | | 0.80 | | 21.10 | | | 0.39 | | 77.38 | | | 59.55 | | | 7.83 | | 3.25 | |
|  | SMF R 367 | right d2 | 138.50 | 18.77 | 126.00 | | 15.30 | | 12.50 | | 6.10 | | | 90.97 | 28.04 | | 2.44 | | | 21.53 | | 1.95 | | | 44.09 | | 4.50 | | | 5.86 | | 0.98 | | |
|  | SMF R 359 | right p2 | 116.67 | 16.82 | 107.00 | 20.46 | | | 9.67 | 3.68 | | | | 91.71 | 26.92 | 1.74 | | | | 20.97 | 1.60 | | | | 52.65 | 1.29 | | | | 4.75 | | 0.40 | | |
|  | SMF R 492 | d4 | 123.00 | 6.52 | 109.75 | 5.12 | | | 13.25 | 3.34 | | | | 89.23 | 25.10 | 1.41 | | | | 18.85 | 1.09 | | | | 43.24 | 5.21 | | | | 4.28 | | 0.67 | | |
|  | SMF R 496 | No. 3 | 108.00 | 10.56 | 90.00 | 7.84 | | | 18.00 | 4.95 | | | | 83.33 | 25.61 | 1.01 | | | | 20.63 | 1.00 | | | | 134.23 | 16.33 | | | | 2.65 | | 0.89 | | |
|  | SMF R 4110 | right d4 | 125.25 | 13.46 | 105.50 | 8.17 | | | 19.75 | 5.40 | | | | 84.23 | 25.98 | 0.71 | | | | 21.28 | 0.79 | | | | 131.59 | 8.15 | | | | 2.59 | | 0.38 | | |
|  | SMNS 55751 | right p2 | 109.75 | 16.84 | 106.00 | 14.73 | | | 3.75 | 2.49 | | | | 96.58 | 25.63 | 1.83 | | | | 21.29 | 1.51 | | | | 150.79 | 99.60 | | | | 1.93 | | 1.22 | | |
|  | SMF R 366 | No. 1 | 101.50 | 8.02 | 85.75 | 5.80 | | | 15.75 | 4.55 | | | | 84.48 | 27.98 | 0.96 | | | | 23.67 | 0.99 | | | | 132.95 | 19.03 | | | | 2.35 | | 0.50 | | |
|  | SMF R 4038 | left p2 | 82.33 | 11.56 | 77.00 | 7.79 | | | 5.33 | 3.86 | | | | 93.52 | 29.69 | 1.48 | | | | 25.94 | 1.22 | | | | 102.18 | 72.44 | | | | 1.12 | | 0.80 | | |
|  | SMF R 364 | No. 2 | 113.75 | 8.53 | 107.50 | 9.55 | | | 6.25 | 4.02 | | | | 94.51 | 27.87 | 0.51 | | | | 24.02 | 0.85 | | | | 150.29 | 25.12 | | | | 2.34 | | 0.52 | | |
| *Psephoderma alpinum* | BSP 1964 XVII 26 | d2 | 284.75 | 44.46 | 175.75 | 23.27 | | | 109.00 | 21.60 | | | | 61.72 | 21.73 | 0.80 | | | | 15.66 | 0.63 | | | | 53.36 | 3.88 | | | | 2.08 | | 0.22 | | |
|  | PIMUZ A/III1255 | isolated | 242.33 | 12.76 | 161.00 | 7.35 | | | 81.33 | 12.04 | | | | 66.44 | 17.23 | 0.46 | | | | 12.51 | 0.34 | | | | 92.57 | 13.54 | | | | 2.15 | | 0.31 | | |
|  | PIMUZ A/III4525 | isolated | 225.00 | 46.05 | 159.25 | 27.85 | | | 65.75 | 20.30 | | | | 70.78 | 17.95 | 0.66 | | | | 13.62 | 0.76 | | | | 101.07 | 5.79 | | | | 1.96 | | 0.30 | | |
| *Henodus chelyops* | GPIT RE 7292 (IV) | right d | 389.00 | 21.00 | 171.00 | 5.00 | | | 218.00 | 16.00 | | | | 43.96 | 12.87 | 1.69 | | | | 8.94 | 1.10 | | | | 69.62 | 1.40 | | | | 1.51 | | 0.06 | | |
|  | GPIT RE 7290 (II) | right d | 416.33 | 42.46 | 199.00 | 20.40 | | | 217.33 | 23.44 | | | | 47.80 | 14.09 | 0.55 | | | | 10.69 | 0.62 | | | | 77.55 | 7.58 | | | | 1.46 | | 0.12 | | |
| *Placochelys placodonta* | SZTFH Ob.2323, Vt.3 | fragmented mand. (d2) | 231.25 | 10.30 | 127.50 | 15.53 | | | 103.75 | 11.23 | | | | 55.14 | 21.87 | 0.94 | | | | 17.13 | 0.54 | | | | 99.86 | 3.49 | | | | 1.64 | | 0.08 | | |
|  | SZTFH Ob.2323, Vt.3 | isolated | 276.75 | 30.19 | 140.75 | 10.92 | | | 136.00 | 20.96 | | | | 50.86 | 18.91 | 1.02 | | | | 14.61 | 0.86 | | | | 83.98 | 5.47 | | | | 1.86 | | 0.07 | | |
|  | SZTFH Ob.2323, Vt.3 | holotype skull (left p2) | 432.33 | 10.87 | 252.00 | 4.97 | | | 180.33 | 15.80 | | | | 58.29 | 16.56 | 0.17 | | | | 12.02 | 0.17 | | | | 77.06 | 6.10 | | | | 1.92 | | 0.17 | | |
| *Paraplacodus broilii* | PIMUZ uncatalogued | II/No.3 | 205.00 | 37.48 | 117.67 | 40.61 | | | 87.33 | 9.53 | | | | 57.40 | 22.66 | 2.63 | | | | 15.54 | 0.93 | | | | 92.87 | 1.98 | | | | 1.95 | | 0.16 | | |
|  | PIMUZ T 4776 | I/No.I | 255.00 | 26.95 | 129.50 | 10.23 | | | 125.50 | 18.58 | | | | 50.78 | 22.94 | 0.29 | | | | 15.64 | 0.59 | | | | 86.18 | 7.14 | | | | 2.69 | | 0.20 | | |
|  | PIMUZ T 5927 | p3 | 327.25 | 20.57 | 158.00 | 9.19 | | | 169.25 | 23.18 | | | | 48.28 | 21.87 | 1.20 | | | | 15.33 | 1.11 | | | | 78.40 | 4.96 | | | | 2.32 | | 0.37 | | |
|  | PIMUZ T 2805 | left p1 | 244.00 | 1.00 | 129.00 | 10.00 | | | 115.00 | 11.00 | | | | 52.87 | 21.98 | 1.57 | | | | 16.36 | 1.50 | | | | 81.32 | 3.24 | | | | 2.47 | | 0.02 | | |
|  | MTM VER 2019.124. | mx2 | 319.00 | 13.32 | 154.50 | 14.04 | | | 164.50 | 5.72 | | | | 48.43 | 19.98 | 1.05 | | | | 14.60 | 0.92 | | | | 82.61 | 1.39 | | | | 2.04 | | 0.35 | | |
| *Macroplacus raeticus* | BSP 1967 I 324 | left p1 | 42.00 | 4.00 | 39.00 | 1.00 | | | 3.00 | 3.00 | | | | 92.86 | 46.94 | 0.49 | | | | 38.19 | 1.43 | | | | 32.39 | 32.39 | | | | 2.17 | | 2.17 | | |
| *Dugong dugon* | MTM.64.67.1 | left mx2 | 312.33 | 25.11 | 78.67 | 20.74 | | | 233.67 | 12.50 | | | | 25.19 | 15.39 | 0.32 | | | | 10.85 | 0.20 | | | | 113.14 | 3.84 | | | | 1.51 | | 0.02 | | |
| *Trichechus manatus* | MTM.64.68.1 | left d1 | 306.33 | 19.87 | 118.33 | 26.64 | | | 188.00 | 23.42 | | | | 38.63 | 12.91 | 0.15 | | | | 8.83 | 0.31 | | | | 118.11 | 9.17 | | | | 1.77 | | 0.01 | | |
| *Enhydra lutris* | MTM.87.67.1 | right mx3 | 271.50 | 45.29 | 148.25 | 19.97 | | | 123.25 | 27.00 | | | | 54.60 | 22.13 | 0.58 | | | | 16.66 | 0.38 | | | | 99.53 | 1.36 | | | | 2.38 | | 0.12 | | |
|  | MTM.2020.35.1 | right mx3 | 252.50 | 12.85 | 126.00 | 17.16 | | | 126.50 | 14.67 | | | | 49.90 | 20.83 | 0.42 | | | | 14.90 | 0.31 | | | | 104.58 | 1.74 | | | | 3.07 | | 0.06 | | |
|  | MTM.64.53.1 | right mx3 | 260.25 | 26.38 | 162.75 | 21.05 | | | 97.50 | 7.53 | | | | 62.54 | 20.62 | 0.50 | | | | 15.35 | 0.37 | | | | 86.57 | 1.70 | | | | 3.10 | | 0.10 | | |

* Age and site data of each specimens of *Cyamodus* sp.:

SMNS 15990: *Data is missing.*

SMNS 81181: Ober Muschelkalk (Anisian-Ladinian boundary); Trebgast (Hegnabrunn, Germany)

SMNS 91472: Trochitenkalk (Anisian); Schopfloch (Germany)

SMNS 56506: *Age data is missing.* ; Niedenfels (Germany)

SMF R 5030: *Data is missing.*

SMF R 5037: *Data is missing.*

SMF R 4040: Ober Muschelkalk (Anisian-Ladinian boundary); Bayreuth (Germany)

SMF R 4039: Ober Muschelkalk (Anisian-Ladinian boundary); Bayreuth (Germany)

SMF R 5028: *Data is missing.*

MTM VER 2013.15: Late Ladinian; Somssich Hill – Villány Mts. (Hungary)

MTM VER 2019.15: Late Ladinian; Somssich Hill – Villány Mts. (Hungary)
