# Supplementary material for: Complex dental wear analysis reveals dietary shift in Triassic placodonts (Sauropsida, Sauropterygia)
Source: Swiss J Palaeontol. 2024 Feb 5;143(1):4. doi: 10.1186/s13358-024-00304-x (PMC10844150; doi:10.1186/s13358-024-00304-x)
Supplement: Supplementary file 2 — Additional file 2. Results of 3D microwear analysis for all specimens. [file 13358_2024_304_MOESM2_ESM.docx]

**Supplementary 2** Results of 3D microwear analysis. Abbreviations: Asfc=complexity, Smc=scale of maximum complexity, HAsfc9= heterogeneity of area-scale fractal complexity, epLsar=anisotropy, SD=standard deviation, d=dentary tooth, mx=maxilla tooth, p=palatine tooth.

| **Taxa** | **Inventory number** | **Element/tooth** | **Asfc** | | **Smc (µm)** | | | | **HAsfc9** | | | | **epLsar** | | | |
| --- | --- | --- | --- | --- | --- | --- | --- | --- | --- | --- | --- | --- | --- | --- | --- | --- |
|  |  |  | mean | SD | mean | | SD | | mean | | SD | | mean | | SD | |
| *Placodus gigas* | SZTFH V.15164 | d3 | 1.5512 | 0.4033 | 9.7211 | | 1.8083 | | 0.5254 | | 0.1833 | | 0.0183 | | 0.00040506 | |
|  | SMF R 361 | left p1 | 1.3536 | 0.3227 | 12.7117 | | 2.8358 | | 0.3639 | | 0.1566 | | 0.0180 | | 0.00028254 | |
|  | SMF R 362 | left p2 | 0.5963 | 0.0903 | 12.8508 | | 0.3510 | | 0.5174 | | 0.1722 | | 0.0175 | | 0.00037177 | |
|  | SMF R 366 | No. 1 | 1.4269 | 0.5193 | 9.5775 | | 0.5311 | | 0.5094 | | 0.1665 | | 0.0182 | | 0.00027446 | |
|  | SMF R 4110 | right d4 | 2.5633 | 1.0113 | 10.2313 | | 1.0787 | | 0.4362 | | 0.1244 | | 0.0186 | | 0.00016069 | |
|  | SMF R 492 | d4 | 0.9028 | 0.1056 | 12.3707 | | 1.5998 | | 0.3712 | | 0.1017 | | 0.0179 | | 0.00023493 | |
|  | SMF R 496 | No. 3 | 1.7445 | 1.4814 | 8.7371 | | 0.6437 | | 0.3137 | | 0.1428 | | 0.0183 | | 0.00041416 | |
|  | SMNS 55751 | right p2 | 1.2194 | 0.1601 | 9.2708 | | 0.0000 | | 0.2031 | | 0.0258 | | 0.0177 | | 0.00015616 | |
| *Cyamodus hildegardis* | PIMUZ T 2796 | left p2 | 0.6616 | 0.1525 | 11.7813 | | 3.3801 | | 0.3009 | | 0.2511 | | 0.0181 | | 0.00006656 | |
| *Paraplacodus broilii* | MTM VER 2019.124. | mx2 | 3.3769 | 0.4561 | 9.2708 | | 0.0000 | | 0.3316 | | 0.1430 | | 0.0177 | | 0.00032282 | |
|  | PIMUZ uncatalogued | II/No.3 | 7.9393 | 4.2354 | 19.1325 | | 13.0158 | | 0.6701 | | 0.3520 | | 0.0173 | | 0.00027288 | |
|  | PIMUZ T 4776 | I/No.I | 1.8146 | 0.7315 | 9.4310 | | 1.7851 | | 0.5215 | | 0.1342 | | 0.0185 | | 0.00032048 | |
|  | PIMUZ T 5927 | p3 | 3.5189 | 1.1734 | 171.3232 | | 228.3099 | | 0.6929 | | 0.6241 | | 0.0181 | | 0.0004827 | |
| *Cyamodus* cfr. *rostratus* | SMNS 17403 | left p2 | 1.7380 | 0.5755 | 10.7690 | 1.5222 | | | 0.3729 | | 0.0510 | | 0.0180 | | 0.00012586 | |
| *Cyamodus kuhnschnyderi* | SMNS 15855 | right p2 | 4.7409 | 0.9742 | 8.5975 | 0.5691 | | | 0.3548 | | 0.0759 | | 0.0179 | | 0.00012925 | |
|  | SMNS 16270 | left p3 | 4.0705 | 0.9633 | 9.0438 | 1.0118 | | | 0.3789 | | 0.1507 | | 0.0185 | | 0.00033975 | |
| *Cyamodus* sp. | MTM VER 2013.15. | isolated | 0.6435 | 0.1226 | 14.5916 | 1.1221 | | | 0.4955 | | 0.0148 | | 0.0180 | | 0.00052741 | |
|  | MTM VER 2019.15. | isolated | 1.6590 | 0.5228 | 15.9643 | 2.7172 | | | 0.3805 | | 0.0964 | | 0.0180 | | 0.00010352 | |
|  | SMF R 4040 | left d4 | 1.6815 | 0.5708 | 12.9556 | 2.8397 | | | 1.1292 | | 0.3779 | | 0.0182 | | 0.00023723 | |
|  | SMF R 5028 | isolated | 2.7297 | 1.1150 | 9.1312 | 0.2418 | | | 0.4687 | | 0.0652 | | 0.0184 | | 0.00036209 | |
|  | SMF R 5030 | isolated | 1.7916 | 2.0781 | 16.4678 | 2.8899 | | | 1.0337 | | 0.3328 | | 0.0182 | | 0.0007372 | |
|  | SMNS 15990 | isolated | 1.7327 | 0.3943 | 17.8713 | 3.3432 | | | 0.7893 | | 0.4852 | | 0.0178 | | 0.00055043 | |
|  | SMNS 81181 | isolated | 1.9209 | 1.1271 | 13.8374 | 1.6820 | | | 0.5703 | | 0.2669 | | 0.0179 | | 0.00033676 | |
|  | SMNS uncatalogued | isolated | 2.0945 | 0.8445 | 9.9247 | 1.1325 | | | 0.5804 | | 0.1842 | | 0.0184 | | 0.00027892 | |
| *Placochelys placodonta* | SZTFH Ob.2323, Vt.3 | isolated | 2.1062 | 0.4660 | 10.3995 | 1.1567 | | 0.6914 | | 0.1875 | | 0.0179 | | 0.0003278 | |  |
|  | SZTFH Ob.2323, Vt.3 | fragmented mand. (d2) | 1.8004 | 0.4918 | 9.1312 | 0.2418 | | 0.4876 | | 0.0825 | | 0.0176 | | 0.0003329 | |  |
| *Henodus chelyops* | GPIT RE 7290 (II) | right d | 1.3389 | 0.1974 | 15.7420 | 2.0408 | | 0.3634 | | 0.0658 | | 0.0184 | | 0.00033795 | |  |
| *Psephoderma alpinum* | BSP 1964 XVII 26 | d2 | 2.1718 | 0.3262 | 12.3665 | 1.6842 | | 0.3984 | | 0.0981 | | 0.0175 | | 0.00024563 | |  |
|  | PIMUZ A/III1255 | isolated | 0.7645 | 0.1821 | 9.5705 | 1.1845 | | 0.3563 | | 0.0855 | | 0.0187 | | 0.00037972 | |  |
| *Macroplacus raeticus* | BSP 1967 I 324 | left p1 | 3.1547 | 0.9882 | 15.4025 | 6.9052 | | 0.9544 | | 0.4851 | | 0.0180 | | 0.00049443 | |  |
